# Molybdate Recovery by Adsorption onto Silica Matrix and Iron Oxide Based Composites

**DOI:** 10.3390/gels8020125

**Published:** 2022-02-16

**Authors:** Florin Matusoiu, Adina Negrea, Mihaela Ciopec, Narcis Duteanu, Petru Negrea, Paula Svera, Catalin Ianasi

**Affiliations:** 1 Faculty of Industrial Chemistry and Environmental Engineering, Polytechnic University of Timişoara, Victoriei Square, No. 2, 300006 Timişoara, Romania; florinmatusoiu95@gmail.com (F.M.); mihaela.ciopec@upt.ro (M.C.); narcis.duteanu@upt.ro (N.D.); petru.negrea@upt.ro (P.N.); 2 National Institute for Research and Development in Electrochemistry and Condensed Matter, 144th Dr.A.P. Podeanu Street, 300569 Timişoara, Romania; paulasvera@gmail.com; 3 “Coriolan Drăgulescu” Institute of Chemistry, Bv. Mihai Viteazul, No. 24, 300223 Timişoara, Romania

**Keywords:** molybdate, adsorption, xerogel, composite, silica and iron oxide matrix

## Abstract

Aggressive industrial development over the last century involved different heavy metals being used, including high quantities of molybdenum, which need to be treated before discharge in industrial waters. Molybdenum’s market price and industrial applicability make its recovery a big challenge. In the present study the possibility to recover molybdenum ions from aqueous solutions by adsorption on a composite material based on silica matrix and iron oxides—SiO_2_FexOy—was evaluated. Tests were performed in order to determine the influence of adsorbent material dose, initial solution pH, contact time and temperature over adsorption capacity of synthesized adsorbent material. For better understanding of the adsorption process, the obtained experimental data were modelled using Langmuir, Freundlich and Sips adsorption isotherms. Based on the obtained data, it can proved that the Sips isotherm was describing with better orderliness the studied process, obtaining a maximum adsorption capacity of 10.95 mg ${\mathrm{MoO}}_{4}^{2-}$ for each gram of material. By modelling the studied adsorption process, it was proven that the pseudo-second order model is accurately describing the adsorption process. By fitting experimental data with Weber-Morris model, it was proven that ${\mathrm{MoO}}_{4}^{2-}$ adsorption is a complex process, occurring in two different steps, one controlled by diffusion and the second one controlled by mass transfer. Further, studies were performed in order to determine the optimum pH value needed to obtain maximum adsorption capacity, but also to determine which are the adsorbed species. From pH and desorption studies, it was proven that molybdate adsorption is a physical process. In order to establish the adsorption mechanism, the thermodynamic parameters (ΔG0, ΔH0 and ΔS0) were determined.

## 1. Introduction

Molybdenum is well known for its biological role, but also due to its importance in the nitrogen cycle in plants metabolism. Molybdenum is considered an essential oligo-element for human beings, being an enzymatic cofactor for several enzymes. In this context, a daily intake between 75 and 250 mg is recommended for adults [1].

In nature, molybdenum can be found only in combined form in different minerals, having different oxidation numbers. The main source of molybdenum is represented by molybdenite (molybdenum sulphide) [2]. Due to its bioaccumulation and high stability, it can be found in small quantities in sea water. Anionic form of molybdenum is easily included into the fodder plants structure, from where it can be accumulated into ruminants during grazing [3].

In a free state, it is a silver metal able to easily form stable and hard carbides. When added in lower quantities improves the hardness, corrosion resistance and elasticity of steel, being used for preparation of high resistant steels. Additionally, molybdenum was used for preparation of different protective layers [4], and special mirrors [5,6], also being used for construction of planes and rockets. In petrochemical plants, catalysts containing alumina and different metals, such as molybdenum, nickel and cobalt, are used for desulphurization operations and for hydro-treating of petroleum heavy fractions [7,8]. In the transistors and solar cells production is used as thin intermediary layer for photovoltaic panels [9], and also as an electrical conductor for electrical contacts, being used for production of halogen lamps, and Rontgen lamps used in medical field, withal.

In recent years, due to the extensive usage, a molybdenum deficit was reported all over the world [3,10,11], making it important to recover it from all secondary resources. Industrial wastewaters contain important quantities of molybdenum, making their treatment necessary before discharge.

A possible way to recover molybdenum from spent HTC catalysts is by pyro metallurgical and hydro-metallurgical processes [7,8,12,13,14,15]. Such recovery processes start with thermal pre-treatment when sulphur and coke are removed, concomitant with the transformation of Mo, Ni, Co, and V in oxides. The further obtained product is leached using sodium hydroxide, to recover Na_2_MoO_4_—a water soluble compound. Other technologies used for molybdenum recovery consist in acidic lixiviation of spent catalysts when is obtained a liquid solution containing Mo, Ni, Co, V and Al [8]. Other technologies that can be used for molybdenum recovery from industrial wastewaters are: selective precipitation [15,16,17], solvent extraction [17,18,19,20,21,22], adsorption on synthetic resins [23,24,25,26,27] and adsorption on activated carbon [15,28]. In some cases, molybdenum recovery can be done by filtration, coagulation, reverse osmosis, and bioremediation. Technologies as reverse osmosis represent a very efficient technology, but it is an expensive one, some technologies as chemical precipitation or solvent extraction are suitable when wastewaters contain relatively high quantities of molybdenum. However, such technologies present a high disadvantage because they generate higher quantities of sludge. Ionic exchange and adsorption represent two promising recovery technologies able to be applied in case of molybdenum. From these two techniques, adsorption represents the most used one due to its simplicity, and higher efficiency correlated with the possibility to easily recover adsorbed metallic ion. An ideal adsorbent material must have mechanical stability, higher adsorption capacity and must be easy to regenerate [29]. Several adsorbents were proposed to be used for molybdate recovery as anions from aqueous solutions [30,31], such as: lignocellulose [32], gold [33,34], silver [35], iron [36], natural minerals as zeolites [37,38,39], calcite [40], bentonite [29], goethite [40,41,42,43,44,45,46], organo-kaolinite [47], graphene oxide [48], metallic hydroxides or metallic oxides [49]. Metallic oxides are characterized by the presence of different active groups on the surface, so such adsorbent materials present a higher adsorption capacity [50], being extensively used for adsorption of metallic and non-metallic ions from waste waters [51].

The aim of the present study was to prepare and characterize a new adsorbent material, which was further tested for recovery of molybdenum ions by adsorption from aqueous solutions. In order to achieve this objective, an adsorbent material has been used with tailored properties based on silica matrix and iron oxides—SiO_2_Fe_x_O_y_.

## 2. Results and Discussion

### 2.1. Material Characterization

The prepared adsorbent material was characterized by using different characterization techniques, such as: scanning electron microscopy (SEM) coupled with dispersive X-ray spectroscopy (EDX), Raman spectroscopy, atomic force microscopy, Fourier transform infrared spectroscopy and thermogravimetric analysis. Spectroscopy methods were used in order to observe the bond interactions and therefore the authenticity of the prepared material. Since Raman and FT-IR methods are complementary, both were used, especially because the presence of some fluorescence in the Raman spectrum. Raman spectroscopy represents an excellent tool for detecting homo-nuclear molecular bonds, whereas FT-IR is sensitive to hetero-nuclear functional group vibrations and polar bonds. After a preliminary analysis of the data obtained from the characterization of the materials indicates that the material has the potential to be a good adsorbent.

#### 2.1.1. Scanning Electron Microscopy (SEM) Coupled with X-ray Dispersive Energy Spectroscopy (EDX)

**Figure 1:**
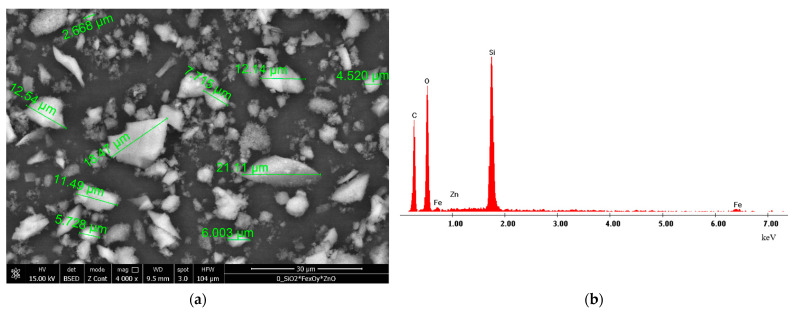
Scanning electron microscopy (SEM) (**a**) şi X-ray dispersive energy spectroscopy (EDX) (**b**) for SiO_2_FexOy material.

In Figure 1 are depicted the recorded SEM micrograph and the recorded EDX spectra for new synthesized adsorbent material.

Analysing the SEM micrograph presented in Figure 1a can be noticed the formation of micrometric size smooth clusters of SiO_2_FexOy, which appears due to the agglomeration of micrometric particles during material’s synthesis. From recorded micrographs was concluded that particles of new prepared adsorbent material have irregular geometrical shapes, being acicular, with dimensions around 10 μm.

From EDX spectra depicted in Figure 1b was determined the amount of chemical elements founded in prepared SiO_2_FexOy, data was depicted in Table 1.

Based on data presented in Table 1 was concluded that the main elements found in new prepared material are C, O, Si and Fe. The presence of C in the sample in this high amount can be associated with the incomplete thermal decomposition of iron acetylacetonate.

#### 2.1.2. Raman Spectroscopy

**Figure 2:**
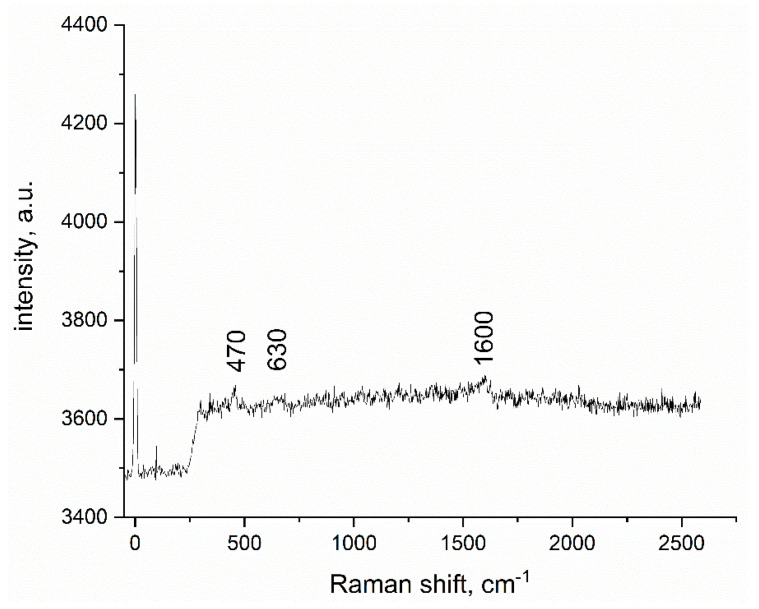
Raman spectra of xerogel sample, SiO_2_FexOy, with laser excitation source (514 nm).

In order to be able to observe vibrational and rotational modes, along with some other low frequency movements, Raman spectroscopy was also used. The recorded spectrum for new prepared adsorbent material—SiO_2_FexOy—is depicted in Figure 2.

Taking into account the composition of the material (Fe(acac)_2_, ammonia and TEOS), several peaks are expected. The presented Raman spectra shows very low intensity peaks around 500 cm^−1^ and 1600 cm^−1^, which are characteristic for the most abundant components, in our case the Fe-O and C-O bonds from the Fe(acac)_2_ [52,53]. Others are expected to be found the Si-O bands, which in our case are overlapped with the already aforementioned bands [52,54].

#### 2.1.3. FT-IR Spectroscopy

**Figure 3:**
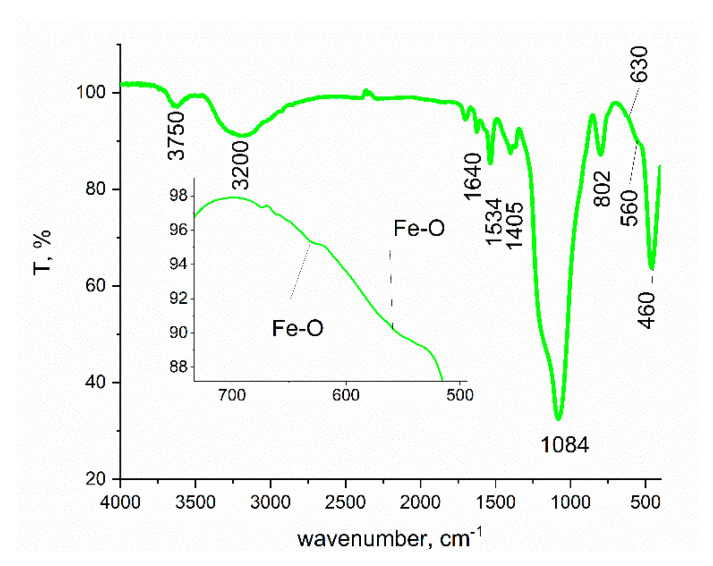
Fourier transform infrared spectroscopy (FT-IR) spectra of xerogel sample, SiO_2_FexOy.

In Figure 3 is depicted the FT-IR spectra recorded for obtained SiO_2_FexOy. Based on determination of the interactions between infrared radiations and matter, it is possible to identify the presence of different bonds due to specific vibrations. Additionally, it is possible to identify different chemical compounds.

In the recorded spectrum can be evidenced the presence of two vibration bands located at 3750 and 3200 cm^−1^, which is associated with the presence of –OH groups. Bands located in the domain 1700–1400 cm^−1^ are specific for the presence of non-decomposed iron acetylacetonate. Additionally, from recorded spectra, there is the presence of the Si-O group, which is associated with the vibrations located at 1640, 1080, 800, and 460 cm^−1^ [55]. The weak band located between 630 and 560 cm^−1^ indicates the presence of Fe-O bonds, being possible to find such a band at 460 cm^−1^, it where can be overlapped with the presence of SiO_2_.

#### 2.1.4. Thermogravimetric Analysis, DTG

**Figure 4:**
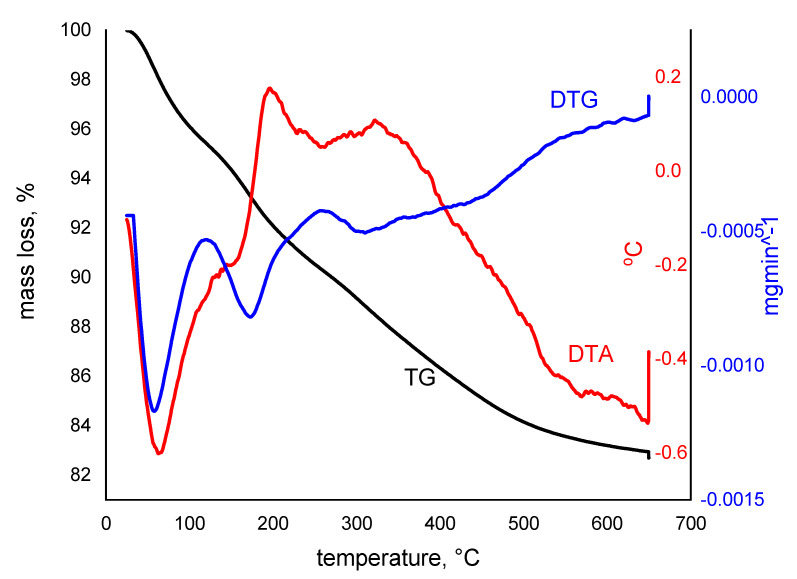
TG, DTG and DTA curves, obtained for thermal degradation of xerogel sample, SiO_2_Fe_x_O_y_, at heating rate of 5 °C min^−1^ in air.

Thermogravimetric analysis has been used to determine the system composition, thermal stability, and oxidative stability of prepared adsorbent material. The thermal decomposition kinetic, evaluation of material corrosive atmosphere, and volatile and water content have also been evaluated. In Figure 4, the recorded TG/DTG/DTA curves for SiO_2_Fe_x_O_y_ are depicted.

Based on recorded curves, a rapid mass loss (around 17%) which takes place in four distinct stages can be observed. Additionally, it was proven that the thermal decomposition is complete around 650 °C. Based on the thermogravimetric curves depicted in Figure 4, this indicates that until 170 °C, concomitant with water loss, the decomposition of iron acetylacetonate (approximatively 5%) will occur, which is an exothermic process. Another exothermic process occurs between 171 and 250 °C, when the thermal decomposition of iron acetylacetonate continues at the same time with loss of small quantities of water and solvents from composite sample (a mass loss around 2%). These exothermic processes can be also associated with the redox reaction between iron II ions acetylacetonate. In the temperature range between 250 and 440 °C, an exothermic process associated with condensation and dihydroxylation of silica is taking place, with a mass loss of around 2%, as well as a thermal decomposition of organic compounds from matrix, and some polymer chain cleavage [56]. At temperature higher than 400 °C can be noticed the presence of another thermal decomposition plateau, associated with 5% mass loss, when the organic residues and C obtained from acetylacetonate decomposition are eliminated. Concomitant can take place the condensation of –OH groups into the silica matrix [56,57,58,59].

Based on the data depicted in Figure 4 and from XRD results (data not shown) was demonstrated that after the xerogel was dried at 100 °C, we succeeded to obtain small quantities of γ-Fe_2_O_3_ due to decomposition of iron acetylacetonate.

#### 2.1.5. Atomic Force Microscopy (AFM)

**Figure 5:**
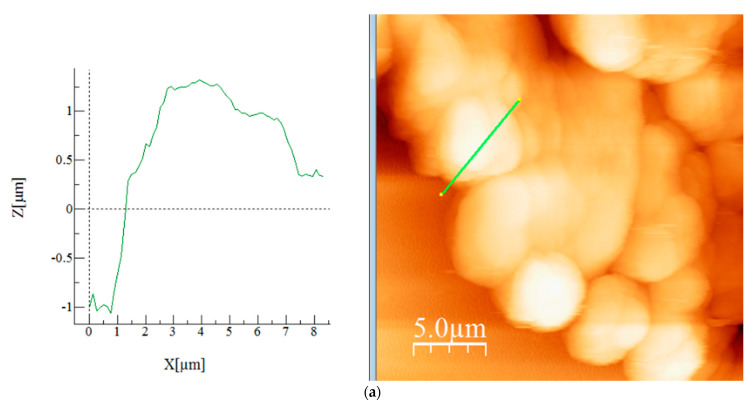

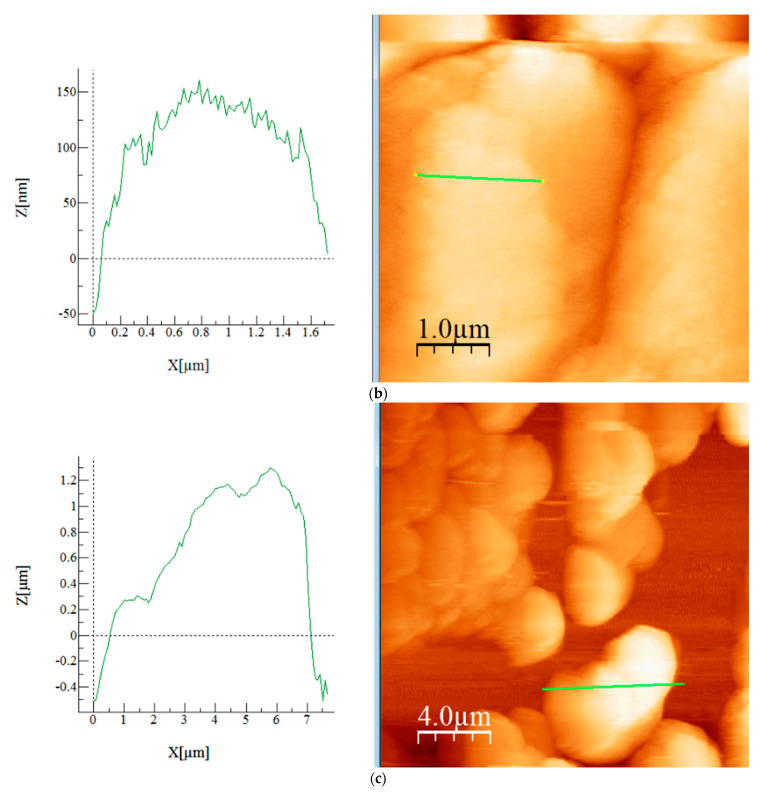

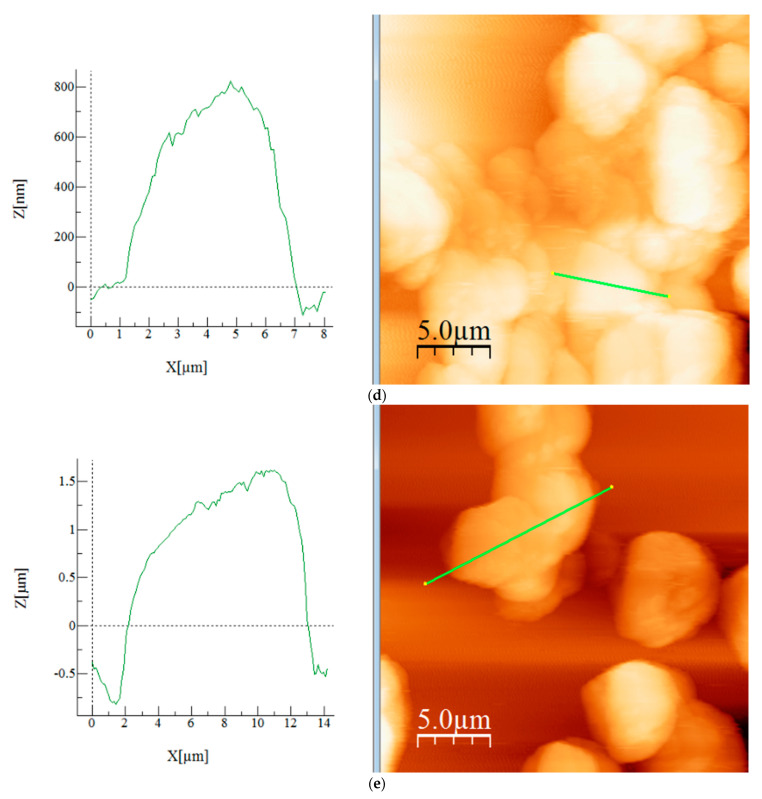

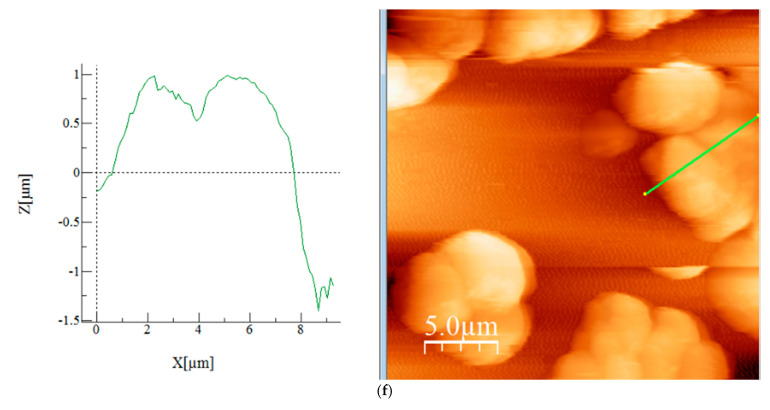
AFM topographical image on a selected area (green line) for sample 1-2 (**a**), 1-7 (**b**), 1-8 (**c**), 1-11 (**d**), 1-13 (**e**) and 1-14 (**f**).

Multiple AFM analyses were performed on the same sample/synthesized material SiO_2_FexOy and the derived images were given name 1-1, 1-7, 1-8, 1-11, 1-13 and 1-14, highlighting the roughness variation on the sample on different areas. The images of the samples were exposed in 2D format (Figure S1a–f from supplementary material) and 3D (Figure S2a–f from supplementary material) format (see supplementary data). The topographical analysis (Figure 5a–f) on the selected areas from 2D images are presented. Roughness data were obtained from the images (Average roughness (Sa), Mean Square Root Roughness (Sq), Maximum peak height (Sp), Maximum valley depth (S), Maximum peak-to-valley height (Sy), Surface kurtosis (Sku), and Surface skewness (Ssk)) and were presented in Table 2.

Topographical images on the selected areas showed the presence of a porous surface which is visible by the asperities on the graphs. Since the analysed sample on 5 × 5 µm is a close-up image, the mentioned asperities are more pronounced. More details regarding this aspect is discussed in the roughness section.

From Table 2, both Sa and Sq parameters are used to evaluate the material’s average roughness difference being the calculation formula [60].

Other important parameter is skewness (Ssk), which indicates the presence of occasional deep valleys or high peaks in the profile as a result of the symmetry measurements of the variation in a profile on the mean line. In this case, both positive skewness (Ssk > 0) and negative skewness (Ssk < 0) were obtained, indicating the presence of high spikes that protrude above the flatter average, respectively, deep valleys below the flatter average [61].

Kurtosis (Sku) is used to describe the density of both high peaks and low valleys, indicating low density when kurtosis has less than 3, and high density when kurtosis has value higher than 3. Sp, Sv and Sy indicate the height of the highest peak value (Sp), the depth of the largest pit (Sv) and their sum (Sy) [62].

Comparing the obtained images (1-2, 1-7, 1-8, 1-11, 1-13, 1-14) it was noticed that they display similar morphology with round structures.

The sample with the highest roughness is 1-13, which also exhibits highest high peak (Sp). This occurrence may be a result of separated particles on a very flat surface which is visible in both 2D and 3D image, where as a consequence the mean line is much lower in comparison to the images where the agglomerates predominate. Still, when comparing the images obtained on the same scale (25 × 25 µm) there are no many differences. As expected, the biggest differences were noticed in case of 20 × 20 µm and 5 × 5 µm scaled images, respectively, in case of sample 1-7 and 1-8.

Regarding the Ssk and Sku values, the Ssk indicates material’s porosity, whereas negative skewness (Ssk < 0) is typical for porous surfaces. The correlation between the Sku and Ssk value can indicate the nature of the surface texturing whereas larger Sku and a more negative Ssk is reflected in more “sliding” surface, with less friction, which is characterized by a wider spacing between the dimples and smaller dimple depths [63]. Still the overall Ssk values are around 0 (and not higher than 1), which confirms the presence of porous surface in case of all analysed samples.

#### 2.1.6. Point of Zero Charge, pH_pZc_

Point of zero charge pH represents the value of pH for specific conditions of temperature, pressure and solution composition, at which the value of the surface charge is zero [63,64]. Zero value of the surface charge means that an equal number of positive and negative charges are found on the surface [65]. Point of zero charge for prepared material has been determined by using the salt addition method, when identical quantities of material were added to a set of electrolyte solutions having the same ionic strength at different pH values [65].

Point of zero charge (pH_pzc_) was determined in order to achieve information regarding SiO2FexOy surface electrical charge. When the pH is higher than pH_pzc_, the material surface will be negatively charged, and if the pH is lower than pH_pzc_, the surface will be positively charged [66,67].

**Figure 6:**
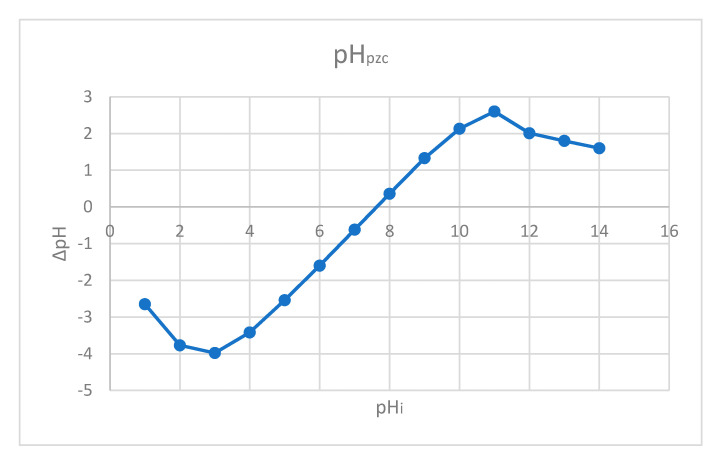
Dependence between initial and final pH of material surface.

In Figure 6 are depicted obtained experimental data regarding the dependence between SiO_2_Fe_x_O_y_ initial and final pH.

Based on data presented in Figure 6, it was found that the zero charge point for new prepared adsorbent material is 7.7. At pH higher than 7.7, the material surface will be negatively charged, leading to some repulsion between material surface and anionic species from solution. At pH lower than 7.7, material surface will be positively charged, favouring anionic species adsorption. By pH increase, material surface will be negatively charged, meaning that the anionic species are rejected [67].

From the data presented in Figure 6 can conclude that at by working at pH lower that 4 will be adsorbed anionic species.

### 2.2. Studies Regarding Molybdate Ion Recovery by Adsorption onto SiO_2_FexOy Material

The adsorption mechanism was established by performing adsorption studies in static regime, following the influence of different parameters, such as: solid:liquid (S:L) ratio, solution pH, contact time, solution temperature, and molybdate initial concentration over the adsorption capacity of new produced adsorbent material.

#### 2.2.1. S:L Ratio Effect

**Figure 7:**
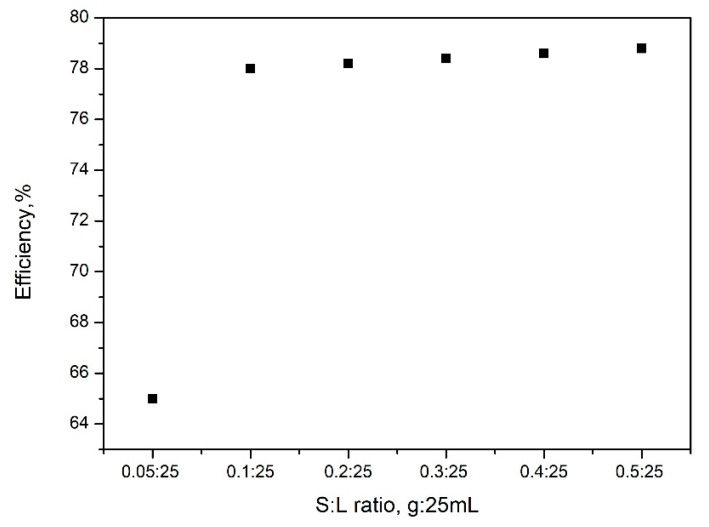
S:L ratio effect on adsorption efficiency.

The obtained experimental data are presented in Figure 7. From the analyses of data presented in Figure 7, it was observed, as was expected, that the ${\mathrm{MoO}}_{4}^{2-}$ adsorption efficiency over SiO_2_FexOy increases with the increase of the S:L ratio. Based on that, it can be said that the adsorption efficiency increases with the increase of S:L ratio, up to a ratio of 0.1 g:25 mL. Any further increase of the ratio S:L leads to at no significant increase of the adsorption efficiency. Based on this observation, any further studies were carried out at a S:L ratio of 0.1:25.

#### 2.2.2. pH Effect

**Figure 8:**
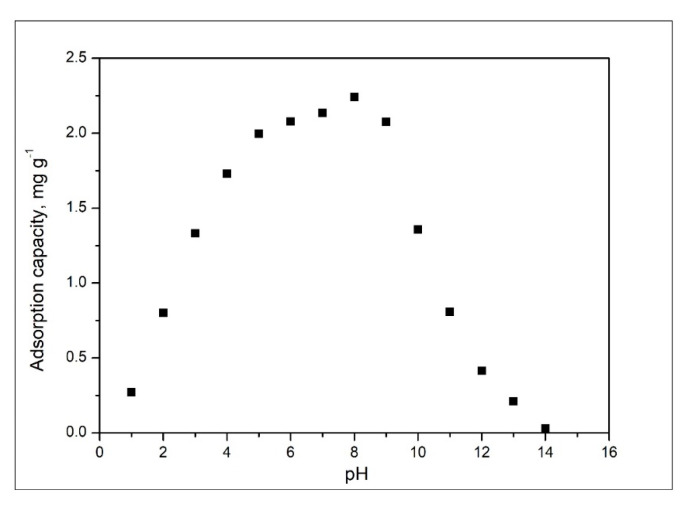
pH effect on adsorption capacity.

Another parameter which affects adsorptive processes is represented by the solution pH. In Figure 8 are depicted obtained experimental data.

By analysing data presented in Figure 6, it is evident that the increase of the solution pH between 1 and 5 leads to an increase of the maximum adsorption capacity. When the pH increase from 6 to 8, the maximum adsorption capacity remains relatively constant, being between 2.08 and 2.24 mg g^−1^. Any further increase of the pH is equivalent to a decrease of the maximum adsorption capacity. Based on obtained data, any further studies were carried out into the pH interval 6 to 8.

**Figure 9:**
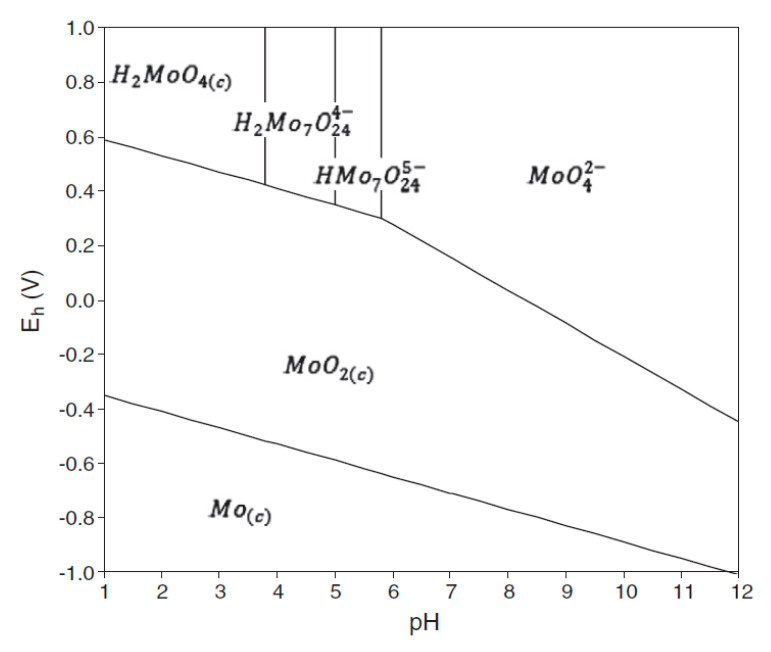
Molybdenum ionic species as a function of pH [8], License No.:5250091421412.

In Figure 9 is presented the dependence between the molybdenum ionic species and the pH. Based on the diagram (depicted in Figure 9) can be observed that the preponderant ionic species into the pH interval 6 to 8 it is ${\mathrm{MoO}}_{4}^{2-}$, meaning that this will be adsorbed onto the new produced adsorbent material.

#### 2.2.3. Contact Time and Temperature Effect

**Figure 10:**
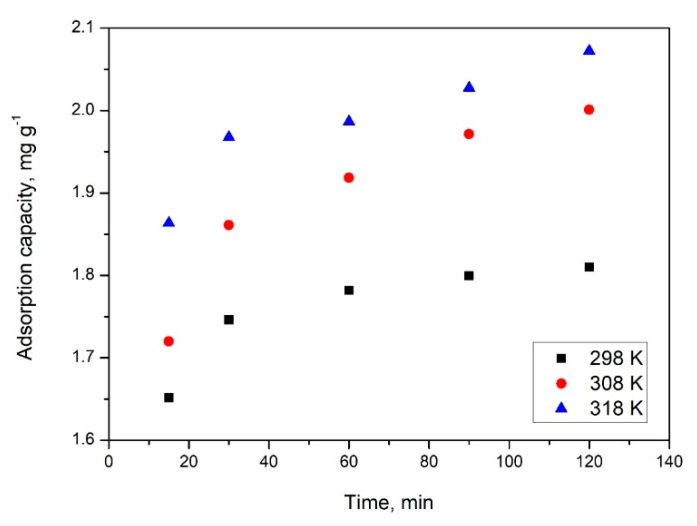
Contact time and temperature influence on adsorption capacity.

Contact time and temperature present an important effect onto the adsorptive processes. Obtained experimental data are presented in Figure 10.

**Figure 11:**
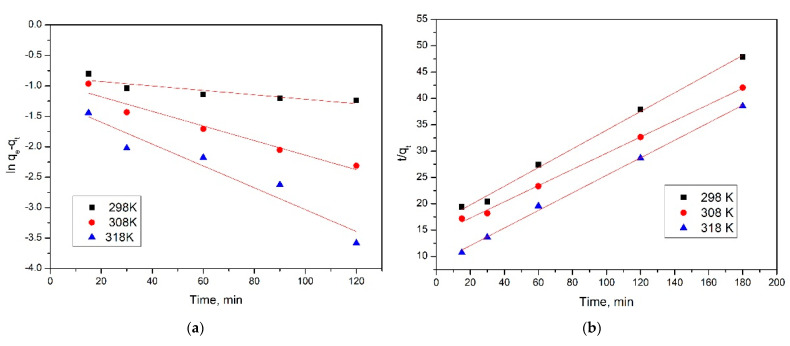
Pseudo-first order (**a**) and pseudo-second order (**b**) isotherms.

From the experimental data presented in Figure 11, it was proven that the increase of the contact time up to 60 min leads at an increase of the SiO_2_FexOy maximum adsorption capacity. By carrying out the adsorptive processes for a contact time higher than 60 min, it can be observed that the maximum adsorption capacity remains relatively constant (1.75–1.98 mg ${\mathrm{MoO}}_{4}^{2-}$ per g of adsorbent material). Based on this observation, it can be concluded that any further adsorption experiments must be carried out for a contact time of 60 min.

Additionally, it is evident that the temperature has a positive effect on maximum adsorption capacity. Therefore, by temperature increase, an increase of the maximum adsorption capacity was obtained, but not a significant one. Based on that, it can be concluded that the adsorption process can be driven at 298 K.

### 2.3. Kinetic Studies

In order to investigate the molybdate adsorption kinetics, the obtained experimental data were modelled using pseudo-first-order and pseudo-second-order models. The kinetic equation used to describe the pseudo-first-order model is [68]:

$$\ln\left(q_{e}-q_{t}\right)=\ln q_{e}-k_{1}t$$

where:

q_e_—equilibrium adsoprtion capacity, mg g^−1^.

q_t_—adsorption capacity at t time, mg g^−1^.

k_1_—speed constant for pseudo-first order equation, min^−1^.

t—contact time, min.

Similarly, the equation which describes the pseudo-second-order model is [69]:

$$\frac{t}{q_{t}}=\frac{1}{k_{2}q_{e}^{2}}+\frac{t}{q_{e}}$$

where:

q_e_—equilibrium adsoprtion capacity, mg g^−1^.

q_t_—adsorption capacity at t time, mg g^−1^.

k_2_—speed constant for pseudo-second order equation, g/mg∙min.

t—contact time, min.

Linear form of pseudo-first-order equation was used for further calculation of adsorption speed constant and predicted adsorption capacity. These data were obtained from the linear dependence between ln(q_e_ − q_t_) versus time (Figure 11a). By using the linear form of the pseudo-second-order equation, we determined the speed constant and predicted adsorption capacity for this model. These data were obtained from graphical representation of the dependence between t/q_t_ versus time (Figure 11b). Calculated values of adsorption speed constants, adsorption capacities, and regression coefficients are presented in Table 3.

From data presented in Table 3, it can be concluded that the studied adsorption process is well-described by pseudo-second-order kinetic model. This conclusion was formulated taking in account the values of the regression coefficient, which are closer to one. The obtained conclusion is also supported by the calculated values of adsorption capacities, which have close values to the experimental obtained one. From the obtained kinetic parameters, it can conclude that the temperature does not have a significant influence, so any further experiments were carried out at 298 K. In addition to the pseudo-first-order and pseudo-second-order kinetic models, the intraparticle diffusion has been studied.

In order to determine if the speed determining stage is the film or intraparticle diffusion, kinetic data were processed using the Weber and Morris model, described by [70]:

q_t_ = k_diff_ × t^1/2^ + C

where:

q_t_—adsorption capacity at t time, mg g^−1^.

k_diff_—speed constant for intraparticle diffusion, mg g^−1^·min^−1/2^.

C—constant correlated with the thickness of the liquid film surrounding the adsorbent particles.

**Figure 12:**
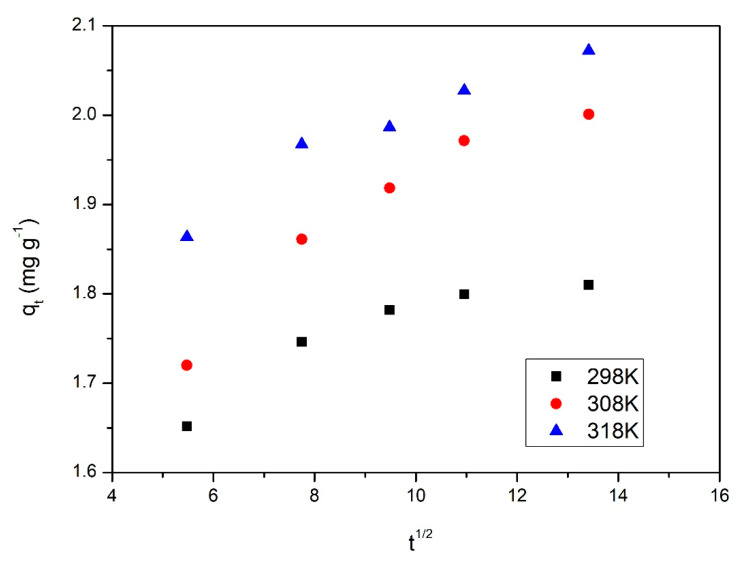
Intraparticle diffusion models for three different temperatures.

In Figure 12 are presented the intraparticle diffusion models obtained at three different temperatures.

From the data presented in Figure 12, it can be concluded that the adsorption of ${\mathrm{MoO}}_{4}^{2-}$ is taking place in several stages, due to the fact that the obtained lines are not going through origin. This means that the kinetics of the studied process are influenced by intraparticle diffusion, but also by film diffusion. The values obtained for speed constant and for film thickness constant are presented in Table 4.

From the data presented in Table 4 was noticed that the diffusion coefficient increases with temperature. Additionally, it can be observed that the diffusion constant specific for first stage of adsorption is higher than the one specific for the second stage of adsorption. Based on that was concluded that the speed determining stage is the second one [71].

Further, from the Arrhenius equation, the value for the activation energy, Ea, was evaluated:

$$\ln k_{2}=\ln A-\frac{E_{a}}{\mathrm{RT}}$$

where:

k_2_—Speed constant, g min^−1^∙mg^−1^.

A—Arrhenius constant, g∙min mg^−1^.

E_a_—Activation energy, kJ mol^−1^.

T—Absolute temperature, K.

R—The ideal gas constant, 8.314 J mol^−1^∙K^−1^.

**Figure 13:**
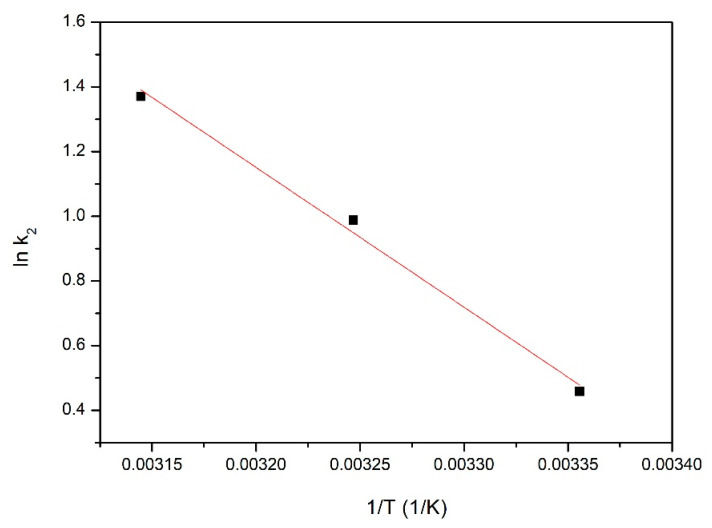
ln k_2_ vs. 1/T plot used for adsorption energy calculation.

During this calculation, the speed constant obtained from the pseudo-second-order kinetic model was used. Activation energy was determined from graphical representation of the dependence between ln k_2_ and 1/T (Figure 13). The activation energy gives us information about the nature of the adsorptive process, proving if the studied process is physical or chemical.

From the data depicted in Figure 13, it was established that the adsorption energy has a value of 19.01 kJ mol^−1^. As the obtained value of the adsorption energy is lower than 40 kJ mol^−1^, the adsorption is a physical one [72].

### 2.4. Thermodynamic Studies

Thermodynamic studies were performed in the temperature range between 298 and 318 K. By using the Gibbs–Helmholtz equation,

$${\Delta G}^{\circ }={\Delta H}^{\circ }-T\cdot {\Delta S}^{\circ }$$

the value for free Gibbs energy was calculated [73].

 where:

ΔG°—standard Gibbs free energy variation, kJ mol^−1^.

ΔH°—standard enthalpy variation, kJ mol^−1^.

ΔS°—standard entropy variation, J mol^−1^∙K^−1^.

T—absolute temperature, K.

**Figure 14:**
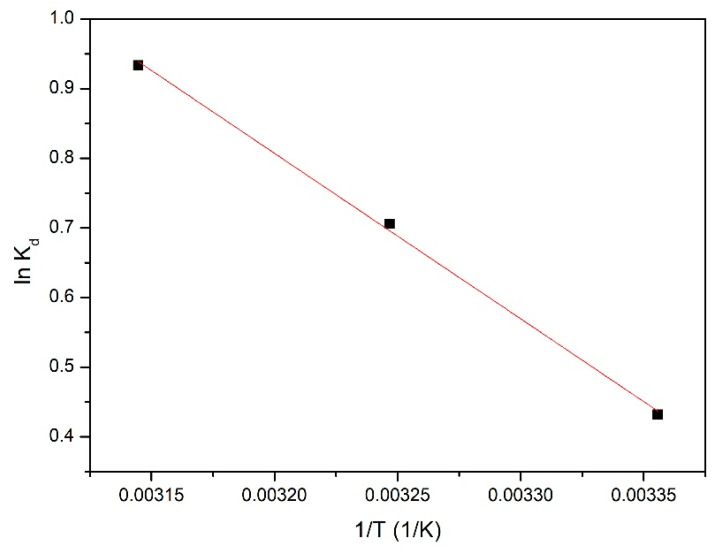
ln K_d_ vs. 1/T plot used for thermodynamic parameters.

From the linear form of the Van ’t Hoff equation (ln K_d_ = f(1/T)) depicted in Figure 14, were calculated the values for standard entropy (ΔS°) and for standard enthalpy (ΔH°).

ln K_d_ = f(1/T)

where:

K_d_—equilibrium constant.

ΔS°—standard entropy variation, J mol^−1^∙K^−1^.

ΔH°—standard enthalpy variation, kJ mol^−1^.

R—the ideal gas constant, 8.314 J mol^−1^∙K^−1^.

Equilibrium constant represents the ratio between the maximum adsorption capacity obtained at equilibrium (q_e_) and equilibrium concentration (C_e_).

Based on the data depicted in Figure 14, we determined the values for the thermodynamic parameters, which are presented in Table 5.

From the data presented in Table 5, it is evident that the standard enthalpy has a positive value, meaning that the studied adsorption is an endothermic one. Additionally, it can be observed that the standard free Gibbs energy has negative values, and increases in absolute value with the increase of the temperature. This behaviour represents a clear indication that the studied adsorption is a spontaneous process, being influenced by temperature. As the value of standard entropy is positive, it was concluded that the adsorption process is a favourable one, which is taking place at the interface solid adsorbent/liquid.

### 2.5. Equilibrium Studies Initial Concentration Effect

**Figure 15:**
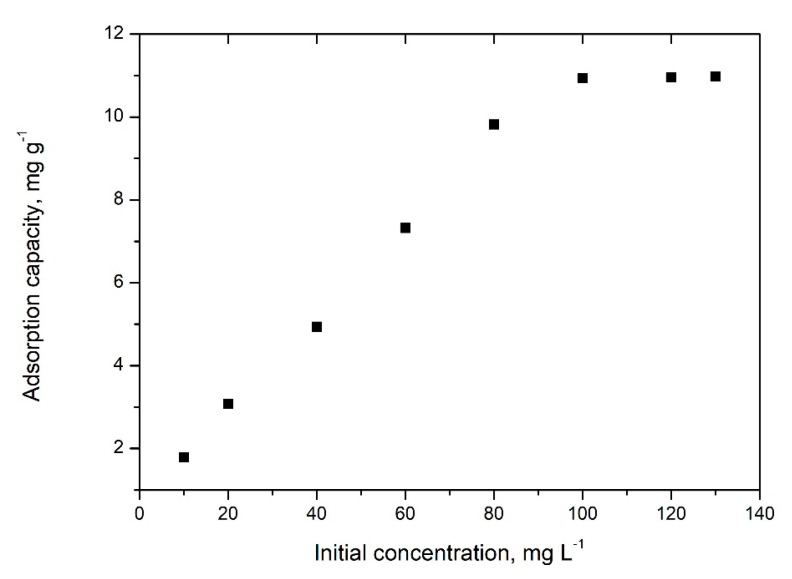
Initial concentration effect on adsorption capacity.

The obtained experimental data regarding the influence of initial concentration during adsorption are presented in Figure 15.

By analysing obtained data, it can be observed that the increase of molybdate initial concentration is equivalent with the increase of the maximum adsorption capacity of new prepared adsorbent material until a plateau is attained. Adsorption capacity of 10.95 mg ${\mathrm{MoO}}_{4}^{2-}$ per gram of adsorbent material was obtained for an initial concentration of 100 mg· L^−1^. Additionally, any further increase of the initial concentration leads to no increase of the adsorption capacity, so we can conclude that this represents the maximum adsorption capacity.

### 2.6. Equilibrium Studies

**Figure 16:**
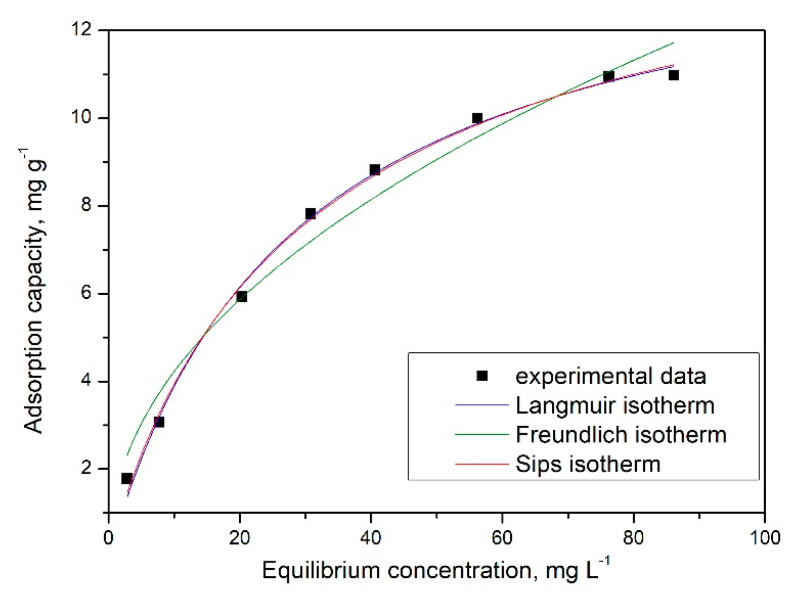
Equilibrium isotherms obtained after experimental data modelling with adsorption isotherm.

Another goal was to establish the adsorption mechanism, when obtained experimental data were modelled using three different adsorption isotherms: Langmuir, Freundlich, and Sips (Figure 16).

Langmuir isotherm considers that adsorbates behave as an ideal gas which is found in the isothermal conditions. This model assumes that the adsorption sites are equivalent, being occupied by only one adsorbate molecule. Linear form of Langmuir isotherm, used for data modelling, is [74]:

$$\frac{C_{e}}{q_{e}}=\frac{1}{q_{L}K_{L}}+\frac{C_{e}}{q_{L}}\;\;$$

where:

q_L_—Langmuir maximum adsorption capacity, mg/g.

K_L_—Langmuir constant.

Freundlich isotherm considers the adsorptive material surface as being a heterogenous one, which is equivalent with an uneven distribution of adsorption heat over the surface. Additionally, this model considers that the adsorption is a multilayer one due to unlimited number of adsorptive centres found on the surface. Linear form of Freundlich isotherm used during modelling is [75]:

$${\mathrm{logq}}_{e}={\mathrm{logK}}_{F}+1/n_{F}{\mathrm{logC}}_{e}\;$$

where:

K_F_ and n_F_—characteristic constants that may be related to the relative adsorption capacity of the adsorbent and the adsorption intensity.

Sips isotherm was derived from Langmuir and Freundlich isotherms [76]. This isotherm presents the Freundlich specific characteristics when the adsorbent concentration is lower, and at higher concentration it is reduced to Langmuir isotherm. Linear from of Sips isotherm used during modelling is:

$$q_{e}=\frac{q_{S}K_{S}C_{e}^{1/n_{S}}}{1+K_{s}C_{e}^{1/n_{S}}}\;$$

unde:

K_S_—constant related to the adsorption capacity of the adsorbent,

n_S_—heterogeneity factor.

Being a combination between Langmuir and Freundlich isotherm, we can consider that: (i) the adsorption process is a homogeneous one, (ii) adsorption is taking place by the interaction between one molecule of solute and one active centre from the adsorbent surface, (iii) sorbent surface have a limited number of active centres, (iv) at equilibrium only a limited number of active centres are occupied, (v) between solute molecules may exist some interactions which allow any further adsorption of solute molecules, and (vi) this model can be used for description of multi-layer adsorption [77].

Based on slopes and from intersection of linear part of the isotherm with y axis, we determined the specific parameters for each used isotherm (Table 6).

From data presented in Table 6, it can be concluded that the adsorption process is better described by Sips isotherm. This conclusion has been reached from analysis of regression coefficient (R^2^), which has a value closer to one, and from the value of maximum adsorption capacity, whose value is closer to the experimentally obtained one.

Based on the data found in scientific literature in Table 7 is presented a comparison between different adsorbent materials used for recovery by adsorption of ${\mathrm{MoO}}_{4}^{2-}$. From the data presented in Table 7, it was concluded that the new produced adsorbent material presents the best adsorption capacity.

### 2.7. Desorption Studies

When the adsorption is carried out by using solutions with pH between 2 and 4, molybdate desorption is reduced (approximately 10%). When the desorption solution has a pH higher than 8, molybdate desorption has a value around 85%. A possible explanation for such behaviour is represented by the presence of the OH^−^ groups. pH effects into the desorption studies confirm that the adsorption process is a physical one. In this context, it can be concluded that only the physically adsorbed ions were desorbed [3].

## 3. Conclusions

In the present study was prepared a new composite adsorbent material in xerogel form, based on SiO_2_FexOy matrix. From obtained experimental data, it was proven that the new produced adsorbent material is efficient for molybdate recovery by adsorption. After preparation, the adsorbent material was characterised by scanning electron microscopy (SEM) coupled with energy dispersive X-ray analysis, Raman spectroscopy, thermogravimetric analysis, FT-IR spectroscopy and atomic force microscopy (AFM). Additionally, the point of zero charge (pH_pZc_) for this new prepared adsorbent material was determined.

During tests, we established the optimum conditions for molybdate recovery by adsorption: liquid—solid ratio—0.1 g:25 mL, pH between 5 and 9, contact time—90 min and temperature of 298 K.

Further, we carried out kinetic, thermodynamic and equilibrium studies, by modelling experimental data. From the kinetic point of view, the studied adsorption is well described by the pseudo-second-order model. In order to determine if intraparticle or film diffusion represent the speed limiting stage, experimental data were modelled using Weber–Moris model.

For SiO_2_FexOy, his porous structure allows the presence of adsorbent sites inside of the adsorbent channels, being an indication that adsorption is taking place in the first stage onto the material surface, and further in the second stage is attained the equilibrium, meaning that the intraparticulate diffusion is not the limiting step. From the value of activation energy, we can conclude that the adsorption is physical.

Based on thermodynamic studies, it was concluded that the studied adsorption is and endothermic one, being spontaneous, influenced by temperature, and taking place on the material surface.

From the equilibrium studies, it was concluded that the process is well described by Sips model, establishing in this way the maximum adsorption capacity (10.95 mg ${\mathrm{MoO}}_{4}^{2-}$ per gram of adsorbent material).

Desorption studies confirmed that the process is controlled by pH, so if the desorption is carried out at pH higher than 8, adsorbent material can be regenerated with higher efficiency.

Based on all that, we can conclude that SiO_2_FexOy can be used for recovery of ${\mathrm{MoO}}_{4}^{2-}$ by adsorption from aqueous solutions.

## 4. Materials and Methods

### 4.1. Material Synthesis and Characterization

Preparation of composite materials based on silicon and iron oxides (SiO_2_Fe_x_O_y_) was realized by using sol gel method. Obtained compound was designed using x and y letters because based on recorded XRD spectrum was proved that the obtained adsorbent material it is an mixture between amorphous silica, Υ-Fe_2_O_3_ and iron (II) acetylacetonate [80]. In this case, a three stage synthesis was used: in the first stage was prepared the silica solution by adding 7 mL of silica precursor (tetra-ethyl orto-silicate—TEOS—Si(OC_2_H_5_)_4_, ≥99.0%, purchased from Sigma-Aldrich Chemie GmbH, Steinheim, Germany) in 37.2 mL of pure ethanol (purchased from SC Chimopar Trading SRL, Bucuresti, Romania, ≥96%). Obtained mixture was mixed for 10 min at 500 rotations per minute (rpm) using a magnetic stirrer. After that, 13.8 mL of water was added, and the obtained mixture was mixed for another 30 min. In second stage was added 0.54 g of iron (II) acetylacetonate (C_10_H_14_FeO_4_—acac, 99.95%, purchased from Sigma-Aldrich Chemie GmbH, Steinheim, Germany), which is used as iron precursor. Obtained mixture was mixed for another 3 h. In third stage, into the reaction mass were added 0.46 g of NH_3_ (purchased from SC Chimopar Trading SRL Bucuresti, Romania, 24%) used as catalyst in order to promote hydrolysis and material condensation. After this stage was obtained a solution with pH 10 before gelation. For xerogel preparation, reaction mass was dried for 24 h at 100 °C, when the thermal decomposition of precursor is taking place. The xerogel sample obtained was named SiO_2_Fe_x_O_y._

Prepared adsorbent material was characterized by scanning electron microscopy (SEM), Thermo Fisher Scientific, Hillsboro, OR, USA, coupled with dispersive X-ray spectroscopy (EDX), Thermo Fisher Scientific, Hillsboro, OR, USA, using a scanning electron microscope FEI Quanta FEG 250, Thermo Fisher Scientific, Hillsboro, Oregon, USA. In order to obtain information about structural footprint, which can be used for further molecules identification, the Raman spectra has been recorded at room temperature using a Shamrock 500i Spectrograph (Oxford Instruments, Andor, UK), equipped with laser excitation source (514 nm). In the next step, the produced material has been analysed by recording the AFM image by using Scanning Probe Microscopy Platform (MultiView-200 system, Nanonics Imaging Ltd., Jerusalem, Israel), using intermittent mode in normal conditions (298 K). This analysis has been carried out by using a chromium-doped tip with a 20 nm radius and 30–40 kHz resonance. Atomic force microscopy (AFM), MultiView-200 system, Nanonics Imaging Ltd., Jerusalem, Israel, is used to quantify the material surface roughness. During AFM analysis, surface images are recorded, and data regarding material characteristic properties such as steps height are obtained. In advance modes, it is possible to determine different physical properties, such as: adherence, dopants distribution, conductivity, surface potential, electrical field, and magnetic areas. For textural proprieties, nitrogen adsorption–desorption measurements for the SiO_2_FexOy were done with Quantachrome Nova1200e apparatus (Anton Paar QuantaTec Inc., Boynton Beach, FL, USA). The surface area of the SiO_2_FexOy obtained by BET method (Brunauer, Emmet, Teller) indicates a value of 305 m^2^/g.

Further, the xerogel was characterized by recording the Fourier transform infrared spectra (FT-IR) by using a JASCO FT/IR-4200 machine (SpectraLab, Shimadzu Corporation, Kyoto, Japan). Derivative thermal analysis (DTG) was performed in order to determine the thermal stability of new produced material. DTG spectra were recorded by using a TGA/SDTA 851-LF Metteler-Toledo system, Mettler-Toledo (HK) MTCN Limtied, Hong Kong, China, between 20 and 650 °C in air atmosphere. In order to fully characterize new prepared material has been determined the zero charge point (pZc) by bringing studied system at equilibrium. In this case were used 0.1 g of SiO_2_Fe_x_O_y_ which was brought in contact with 25 mL KCl 0.1 N. Obtained system has been mixed using a shaker (200 rpm) at 298 K (JUlabo SW23, JULABO GmbH, Seelbach, Germany). Solutions pH was adjusted in range 2 to 12 using NaOH solutions with concentration between 0.05 and 2 N, and HNO_3_ solutions having concentrations between 0.05 and 2 N. After filtration has been measured the pH for each solution by using a Mettler Toledo SevenCompact, S 210 pH metter, Mettler-Toledo (HK) MTCN Limtied, Hong Kong, China.

### 4.2. Studies Regarding Molybdate Ion Recovery by Adsorption onto SiO_2_FexOy Material

#### 4.2.1. Solid:Liquid (S:L) Ratio Effect

To establish which is the optimum S:L ratio, we needed to obtain the best adsorption efficiency. In this case, the quantity of adsorbent material (0.05, 0.1, 0.2, 0.3, 0.4, and 0.5 g of SiO_2_Fe_x_O_y_) was changed for a constant volume (25 mL) of ammonium molybdate ((NH_4_)_6_Mo_7_O_24_ 4H_2_O, purchased from Merck, Darmstadt, Germany) having an initial concentration of 10 mg ${\mathrm{MoO}}_{4}^{2-}$ L^−1^. Adsorptions were carried out using a thermostatic shaker, at temperature of 298 K, for a contact time of 60 min. Adsorption efficiency was determined using the following relation:

$$\mathrm{Efficiency}=\frac{C_{i}-C_{\mathrm{rez}}}{C_{i}}100,\;\left[\%\right]$$

where:

C_i_—${\mathrm{MoO}}_{4}^{2-}$ initial concentration, mg L^−1^.

C_rez_—${\mathrm{MoO}}_{4}^{2-}$ residual concentration, mg L^−1^.

#### 4.2.2. pH Effect

pH influence over adsorption is determined by the form of ${\mathrm{MoO}}_{4}^{2-}$ into the solution and by the nature of SiO_2_Fe_x_O_y_ surface. In the present paper, the influence of the solution pH over the adsorption was studied into the pH range 1 to 14. In this context, 0.1 g of adsorbent were mixed with 25 mL of ${\mathrm{MoO}}_{4}^{2-}$ solution with initial concentration of 10 mg·L^−1^. Obtained mixtures were kept in contact at 298 K for 60 min. pH of the solutions was adjusted using HNO3 and NaOH solutions with concentrations between 0.1 and 1 N. Material adsorption capacity was evaluated using the following relation:

$$q=\frac{\left(C_{i}-C_{\mathrm{rez}}\right)\cdot V}{m},[\mathrm{mg}/g]$$

where:

q—adsorption capacity, mg g^−1^.

C_i_—${\mathrm{MoO}}_{4}^{2-}$ initial concentration, mg L^−1^.

C_rez_—${\mathrm{MoO}}_{4}^{2-}$ residual concentration, mg L^−1^.

V—solution volume, L.

m—SiO_2_Fe_x_O_y_ material mass, g.

#### 4.2.3. Contact Time and Temperature Effect

In order to establish the influence of contact time and temperature over adsorption capacity were weighed samples of 0.1 g, which were mixed with 25 mL solutions of ${\mathrm{MoO}}_{4}^{2-}$, having an initial concentration of 10 mg·L^−1^. Obtained samples were mixed for different time periods (15, 30, 60, and 120 min) at different temperatures (298, 308, and 318 K), at 200 rpm.

#### 4.2.4. Initial Concentration Effect

The effect of initial concentration of ${\mathrm{MoO}}_{4}^{2-}$ ions over maximum adsorption capacity were prepared solutions with initial concentrations of 10, 20, 40, 60, 80, 100, 120, and 130 mg·L^−1^. All adsorptions were carried out into the pH interval between 5 and 9, at 298 K for a mixing time of 90 min.

### 4.3. Desorption Studies

In order to obtain maximum economic efficiency for studied adsorption process, and to establish further the maximum efficiency of prepared adsorbent material, were performed adsorption/desorption studied. Therefore, were weighted 0.1 g of exhausted material, mixed with 25 mL of distilled water, having the pH adjusted into the range 2 to 12, by using NaOH solutions with different concentration (between 1 and 5 N).

Residual concentration of ${\mathrm{MoO}}_{4}^{2-}$ ions was determined by using the atomic adsorption spectrometry, by using a Varian SpectrAA 270 FS spectrometer, Agilent Technologies, Inc., Mulgrave, Victoria, Australia.

## Figures and Tables

**Table 1:** The amount of chemical elements presents in the SiO_2_FexOy material.

Elements	Wt, %	At, %
C K	44.94	54.61
O K	43.53	39.71
Si K	11.14	5.57
Fe K	0.39	0.10
TOTAL	100.00	100.00

**Table 2:** Roughness parameters of sample 1-1, 1-7, 1-8, 1-11, 1-13 and 1-14.

Sample Name	Ironed Area (µm^2^)	Sa (µm)	Sq (µm)	Sp (µm)	Sv (µm)	Sy (µm)	Sku	Ssk
1-2	810.853	0.527	0.652	1.569	−2.353	3.922	2.856	−0.352
1-7	36.054	0.102	0.129	0.372	−0.677	1.049	3.733	−0.788
1-8	537.342	0.344	0.423	1.414	−1.103	2.517	3.122	0.699
1-11	849.807	0.514	0.629	1.436	−3.478	4.914	3.818	−0.565
1-13	894.873	0.642	0.790	3.182	−1.977	5.159	3.078	0.838
1-14	1091.69	0.645	0.762	2.504	−2.054	4.558	2.298	0.130

**Table 3:** Kinetic parameters obtained from data modelling.

Pseudo-First Order				
Temperature (K)	q_e,exp_ (mg g^−1^)	k_1_ (min^−1^)	q_e,calc_ (mg g^−1^)	R^2^
298	1.80	0.004	2.35	0.7919
308	1.97	0.012	2.55	0.9561
318	2.02	0.018	3.44	0.9328
**Pseudo-Second Order**				
**Temperature (K)**	**q_e,exp_** **(mg g^−1^)**	**k_2_** **(g mg^−1^∙min^−1^)**	**q_e,calc_** **(mg g^−1^)**	**R^2^**
298	1.80	1.582	1.18	0.9999
308	1.97	2.686	2.04	0.9999
318	2.02	3.937	2.09	0.9996

**Table 4:** Specific parameters obtained for intraparticle diffusion.

Intraparticle Diffusion Model			
Temperature (K)	K_diff_ (mg g^−1^·min^−1/2^)	C	R^2^
298	0.0194	1.575	0.7874
308	0.0251	1.563	0.8888
318	0.0351	1.747	0.9324

**Table 5:** Thermodynamic parameters.

ΔH°, kJ mol^−1^	ΔS°, J mol^−1^∙K^−1^	ΔG°, kJ mol^−1^			R^2^
19.76	69.96	298 K	308 K	318 K	0.9988
		−1.08	−1.78	−2.48	

**Table 6:** Isotherm parameters obtained based on adsorption isotherm.

Langmuir Isotherm			
q_m,exp_ (mg/g)	K_L_ (L mg^−1^)	q_L_ (mg g^−1^)	R^2^
10.95	0.035	14.85	0.9946
**Freundlich Isotherm**			
**K_F_** **(mg/g)**	**1/n_F_**		**R^2^**
1.415	0.47		0.9696
**Sips Isotherm**			
**K_S_**	**q_S_** **(mg g^−1^)**	**1/n_S_**	**R^2^**
0.038	15.1	0.05	0.9952

**Table 7:** Comparison of adsorption performance with other materials for ${\mathrm{MoO}}_{4}^{2-}$.

Materials	q, mg/g	References
Fe_3_O_4_ incorporated in hydrolysate triazine functionalized polymer	0.213	[78]
Goethite, α-FeO(OH)	1.76	[79]
Hematite, Fe_2_O_3_	1.43	[79]
SiO_2_FexOy	10.95	Present study

## Data Availability

Not applicable.

## Supplementary Materials

The following supporting information can be downloaded at https://www.mdpi.com/article/10.3390/gels8020125/s1, Figure S1. 2D image of sample 1-2, 1-7, 1-8, 1-11, 1-13 and 1-14, Figure S2. 3D image of sample 1-2, 1-7, 1-8, 1-11, 1-13 and 1-14.
