# Supplementary material for: Molybdate Recovery by Adsorption onto Silica Matrix and Iron Oxide Based Composites
Source: Gels. 2022 Feb 16;8(2):125. doi: 10.3390/gels8020125 (PMC8871702; doi:10.3390/gels8020125)
Supplement: Supplementary file 1 [file gels-08-00125-s001.zip › gels-1548565-supplementary.pdf]

gels

MDPI

Supplementary

Molybdate Recovery by Adsorption onto Silica Matrix and Iron Oxide Based Composites

Florin Matusoiu [1], Adina Negrea [1,*], Mihaela Ciopec [1], Narcis Duteanu [1], Petru Negrea [1], Paula Svera [2] and Catalin Ianasi [3,*]

1 Faculty of Industrial Chemistry and Environmental Engineering, Polytechnic University of Timişoara, Victoriei Square, No. 2, 300006 Timişoara, Romania; florinmatusoiu95@gmail.com (F.M.); mihaela.ciopec@upt.ro (M.C.); narcis.duteanu@upt.ro (N.D.); petru.negrea@upt.ro (P.N.)
2 National Institute for Research and Development in Electrochemistry and Condensed Matter, 144th Dr.A.P. Podeanu Street, 300569 Timisoara, Romania; paulasvera@gmail.com
3 "Coriolan Drăgulescu" Institute of Chemistry, Bv. Mihai Viteazul, No. 24, 300223 Timisoara, Romania
* Correspondence: adina.negrea@upt.ro (A.N.); ianasic@acad-icht.tm.edu.ro (C.I.)

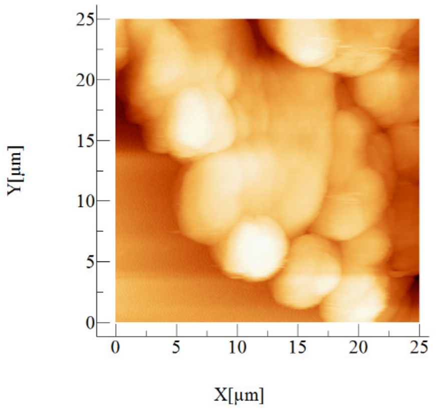

(**a**) 2D image of the sample 1-2 on 25 × 25 μm area

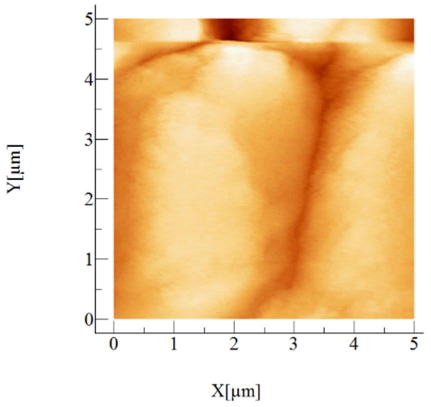

(**b**) 2D image of the sample 1-7 on 5 × 5 μm area

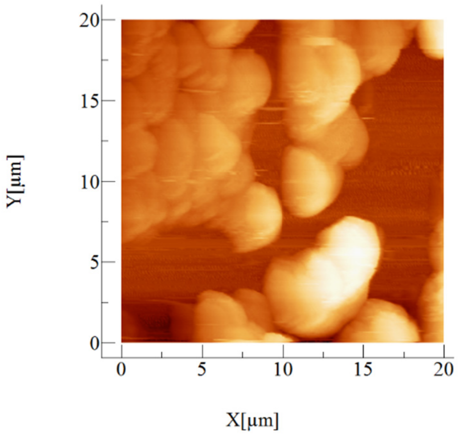

(**c**) 2D image of the sample 1-8 on 20 × 20 μm area

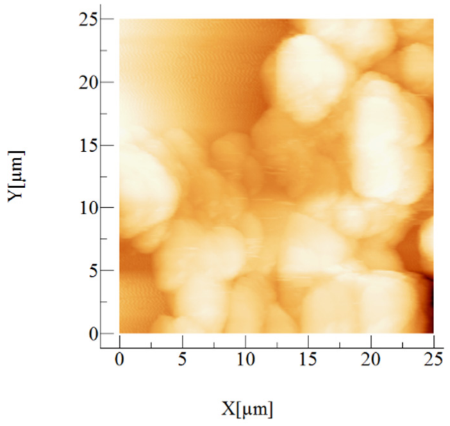

(**d**) 2D image of the sample 1-11 on 25 × 25 μm area

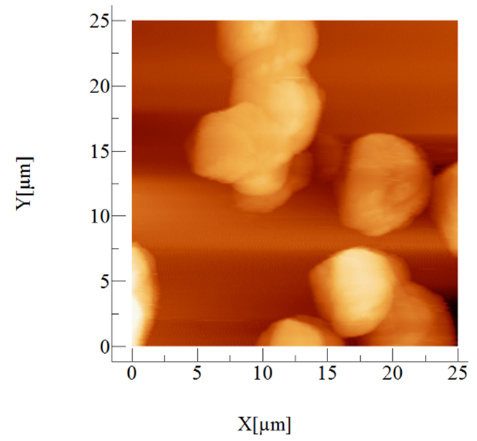

(**e**) 2D image of the sample 1-13 on 25 × 25 µm area

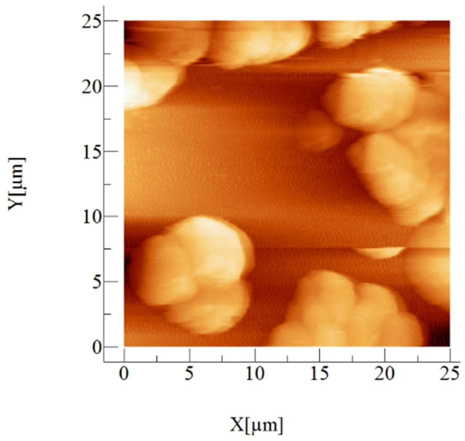

(**f**) 2D image of the sample 1-14 on 25 × 25 µm area

Figure S1. 2D image of sample 1-2, 1-7, 1-8, 1-11, 1-13 and 1-14.

The majority of the analyzed sample areas were performed on 25 × 25 µm region (1-2, 1-11, 1-13 and 1-14), one was performed on 20 × 20 µm region (1-8) and one on 5 × 5 µm region (1-7). The samples on 25 × 25 µm and 20 × 20 µm region resemble, displaying the presence of round shaped particles that are agglomerated on some parts of the images. Regarding the 5 × 5 µm image, less details are visible, since the size of the image contains much smaller area.

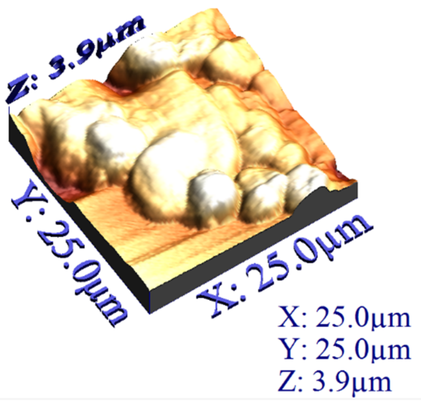

(**a**) 3D image of the sample 1-2 on 25 × 25 µm area

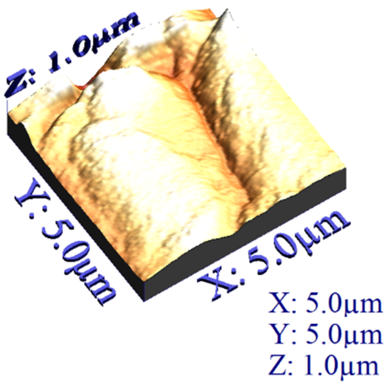

(**b**) 3D image of the sample 1-7 on 5 × 5 µm area

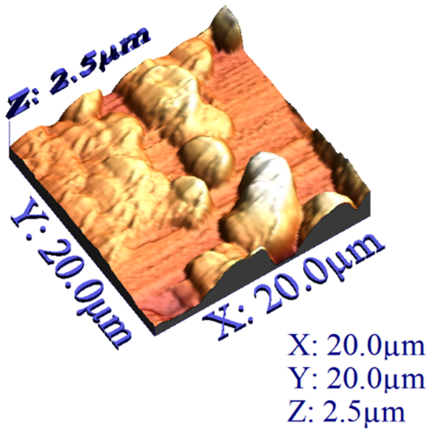

(**c**) 3D image of the sample 1-8 on 20 × 20 µm area

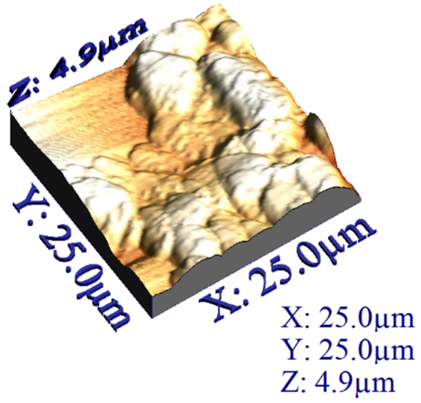

(**d**) 3D image of the sample 1-11 on 25 × 25 µm area

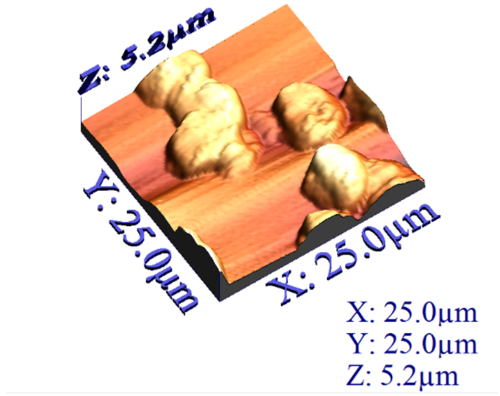

(**e**) 3D image of the sample 1-13 on 25 × 25 µm area

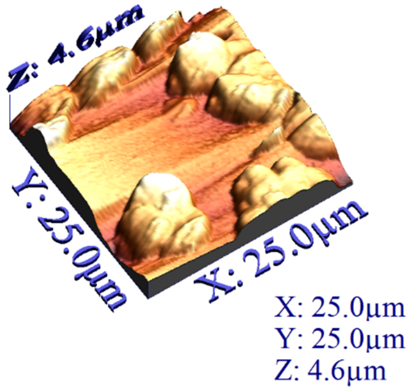

(**f**) 3D image of the sample 1-14 on 25 × 25 µm area

Figure S2. 3D image of sample 1-2, 1-7, 1-8, 1-11, 1-13 and 1-14.

The 3D images give us details regarding the height of the materials, with more visible details that were not seen in the case of 2D images. Highest heights (Z) were observed in case of 1-13 followed by the 1-11, 1-14, 1-2, 1-8 and 1-7.
